# Carfilzomib, lenalidomide, and dexamethasone in relapsed refractory multiple myeloma: a prospective real-life experience of the Regional Tuscan Myeloma Network

**DOI:** 10.3389/fonc.2023.1162990

**Published:** 2023-04-25

**Authors:** Elisabetta Antonioli, Sofia Pilerci, Irene Attucci, Gabriele Buda, Alessandro Gozzetti, Veronica Candi, Federico Simonetti, Maria Livia Del Giudice, Sara Ciofini, Michela Staderini, Sara Grammatico, Alessandra Buzzichelli, Maria Messeri, Monica Bocchia, Sara Galimberti, Alessandro M. Vannucchi

**Affiliations:** ^1^ Haematology Unit, Careggi University Hospital, Florence, Italy; ^2^ Department of Clinical and Experimental Medicine, Hematology, University of Pisa, Pisa, Italy; ^3^ Hematology, Department of Medical Science, Surgery and Neuroscience, University of Siena, Siena, Italy; ^4^ U.O.S Ematologia, San Donato Hospital, Arezzo, Italy; ^5^ U.O.S. Ematologia–Ospedale Versilia, Lido Di Camaiore, Viareggio, Italy; ^6^ S.O.S. Oncoematologia ed Ematologia Clinica, Ospedale Nuovo San Giovanni di Dio – USL Toscana Centro, Florence, Italy; ^7^ S.O.S. Oncoematologia, Ospedale Santo Stefano, Prato, Italy

**Keywords:** multiple myeloma, carfilzomib, real life, clinical trial, prospective observational study

## Abstract

**Introduction:**

Carfilzomib, a potent, irreversible, selective proteasome inhibitor has demonstrated consistent results in relapsed/refractory multiple myeloma (RRMM) combined with lenalidomide and dexamethasone (KRd). No prospective studies are yet available that analyzed the efficacy of the KRd combination.

**Methods:**

Herein, we report a multicenter prospective observational study on 85 patients who were treated with KRd combination as the second or third line of treatment, according to standard practice.

**Results:**

The median age was 61 years; high-risk cytogenetic was found in 26% and renal impairment (estimated glomerular filtration rate (eGFR) <60 ml/min) in 17%. After a median follow-up of 40 months, patients received a median number of 16 cycles of KRd, with a median duration of treatment (DoT) of 18 months (range, 16.1–19.2 months). The overall response rate was 95%, with a high-quality response (≥very good partial remission [VGPR]) in 57% of the patients. The median progression-free survival (PFS) was 36 months (range, 29.1–43.2 months). Achievement of at least VGPR and a previous autologous stem cell transplantation (ASCT) were associated with longer PFS. The median overall survival (OS) was not reached (NR); the 5-year OS rate was 73%. Nineteen patients underwent KRd treatment as a bridge to autologous transplantation, obtaining a post-transplant minimal residual disease (MRD) negativity in 65% of cases. The most common adverse events were hematological, followed by infection and cardiovascular events, rarely G3 or higher, with a discontinuation rate for toxicities of 6%. Our data confirmed the feasibility and safety of the KRd regimen in real life.

## Introduction

In recent years, the clinical outcome of multiple myeloma (MM) patients has improved due to the introduction of several new agents, such as the third-generation immunomodulator (IMiD) pomalidomide, next-generation proteasome inhibitors (PIs) carfilzomib and ixazomib, and the introduction of immunotherapy (daratumumab, isatuximab, and elotuzumab). The combination of novel drugs resulted in improved quality of response, in turn translating into amelioration of progression-free survival (PFS) and overall survival (OS).

Carfilzomib, a second-in-class proteasome inhibitor, was approved in combination with lenalidomide and dexamethasone (KRd; e.g., ASPIRE trial), with dexamethasone alone (Kd, e.g., Endeavor trial) and with anti-CD38 antibody (Isa-Kd, e.g., IKEMA trial; and Dara-KD, e.g., CANDOR trial) for the treatment of patients with MM who have received at least one prior therapy (1–4). In the prospective randomized Phase III ASPIRE trial, carfilzomib, lenalidomide, and dexamethasone (KRd) combination demonstrated an 87.1% overall response rate and a significantly prolonged PFS (26.3 months) when compared with the control arm receiving Rd (2). Subgroup analyses of ASPIRE showed that carfilzomib-containing regimens improve PFS and overall response rate (ORR) regardless of early or late relapse (5) and that PFS and OS were longer for patients who achieved ≥complete remission (CR) (PFS was 50.4 and OS 67.0 months) versus those who achieved a very good partial response or partial response (PFS was 22.1 and OS 44.2 months) (6). Median PFS was also longer for patients with prior autologous stem cell transplantation (ASCT) (26.3 *vs.* 17.8 months, hazard ratio (HR) = 0.68) and in those with a prior line of therapy that included ASCT (7). However, the strict eligibility criteria in randomized clinical trials (RCTs) precluded the inclusion of patients with comorbidities such as renal failure (renal impairment (RI)) and cardiovascular disease (CVD), limiting the generalizability of RCT. In the Chari analysis, up to 72.3% of patients receiving routine care did not meet the eligibility criteria for ASPIRE trial, mainly due to the particularly restrictive specific threshold of renal function (CrCl 50 ml/min for inclusion) (8).

Since then, retrospective real-life investigations have reported the use of the KRd combination in patients with relapsed/refractory multiple myeloma (RRMM) describing its safety and efficacy profiles in heavily pre-treated and very heterogeneous patient populations (9–13). The latest of these, a recent Italian study, reported a joint analysis of 600 RRMM patients treated with KRd, confirming the efficacy of this combination in the real-life context and identifying previous lenalidomide exposure, high-risk cytogenetic alterations, International Staging System (ISS) advanced, and severe renal insufficiency as factors with a negative prognostic impact (14).

However, no prospective studies are yet available that analyze the efficacy of the KRd combination, particularly in early lines of therapy such as in ASPIRE trial. The aim of the present study was to evaluate response and safety data in patients prospectively treated with the KRd regimen in a real-world setting who received one or two prior lines of therapy (LOT).

## Methods

This multicenter prospective observational study was conducted across seven Tuscany hematology centers. It was approved by institutional ethics committees and was conducted in accordance with the principles of the Declaration of Helsinki; all patients gave written informed consent at enrolment. Eighty-five patients were enrolled between 15 December 2016 and 31 December 2019. The patients were then followed up for 2 years (data cutoff December 2021). The only inclusion criteria were a diagnosis of RRMM and a treatment with KRd combination as a second or third line of treatment, according to standard practice. For each patient, we collected baseline data at diagnosis and at the time of carfilzomib therapy initiation, including patient demographics and comorbidities, disease characteristics, renal and bone involvement, the presence of extramedullary disease, and prior therapies. Patients underwent a cardiac echography before the first dose of KRd, and the left ventricular ejection fraction (LVEF) had to be at least ≥50% in all patients. For risk stratification, we used the International Staging System (ISS) (15) and fluorescence *in situ* hybridization (FISH) in CD138+ plasma cells using standard methodology. High-risk FISH was defined when one abnormality among del(17/17p), t(4;14), t(14;16), t(14;20) and amplification(1q) was present. All patients received intravenous K at a standard dose (20 mg/mq on days 1 and 2 of cycle 1 and then 27 mg/mq on days 8, 9, 15, and 16 of the first cycle and days 1, 2, 8, 9, 15, and 16 of the subsequent cycles), in association with dexamethasone (20 mg on days 1, 2, 8, 9, 15, 16, 22, and 23) and lenalidomide (25 mg orally on days 1–21) of each 28-day cycle. According to the ASPIRE schedule, after the 12th cycle, administration of K was reduced (days 1, 2, 15, and 16) and prolonged beyond 18 cycles at the physician’s discretion. Lenalidomide was delivered at 25 mg on days 1 to 21 of each 28-day cycle and dexamethasone at 40 mg weekly. In all patients, antibacterial, antiviral, and antithrombotic prophylaxes were prescribed. The dose of each drug was adjusted, according to drug recommendations, in case of specific pre-existing comorbidities. The lenalidomide starting dose was adjusted according to renal function. Renal impairment (RI) was defined as an estimated glomerular filtration rate (eGFR) ≤50 ml/min. Elderly patients (>75 years) received reduced dexamethasone doses. Treatment was administered until disease progression or unacceptable toxicity. An efficacy assessment was performed on day 1 of each cycle according to the International Myeloma Working Group (IMWG) criteria (16). The overall response rate (ORR) was calculated considering the achievement of at least a PR. In patients with active bone lesions, a skeletal survey was performed by PET/CT at least after 6 months of therapy. To assess the safety and toxicity of treatment, adverse events were described according to the National Cancer Institute Common Terminology Criteria version 5.0 and were collected from the day of treatment initiation through the last dose. We recorded hematological adverse events of grade 3 or higher and non-hematological events, focusing on cardiovascular adverse events (CVAEs), due to the reported relationship between K and cardiac complications (17–19). We also analyzed the need for interruption of therapy for hospitalization, and all the adverse events that required hospitalization were considered serious adverse events (AEs).

### Statistical analysis

All analyses were on an intent-to-treat basis. Statistical analysis was performed using the IBM SPSS Statistics for Windows (version 24.0; Armonk, NY, USA) and R (version 3.5.1) software. Descriptive statistical min, max, mean, and standard deviation (SD) or median and its interquartile range (IQR) were determined for all continuously distributed variables. Categorical variables were described by absolute and relative frequencies. Time to event (progression, death) was calculated from the date of the first dose of KRd; time-to-event curves were plotted with the Kaplan–Meier method, and comparisons among groups were made using the log-rank test. Semi-parametric Cox regression univariate analysis was performed aimed at finding prognostic factors affecting PFS and OS. Cox proportional hazards regression models were used for multivariate analysis. A p-value of less than 0.05 was considered to indicate statistical significance.

## Results

**Figure 1:**
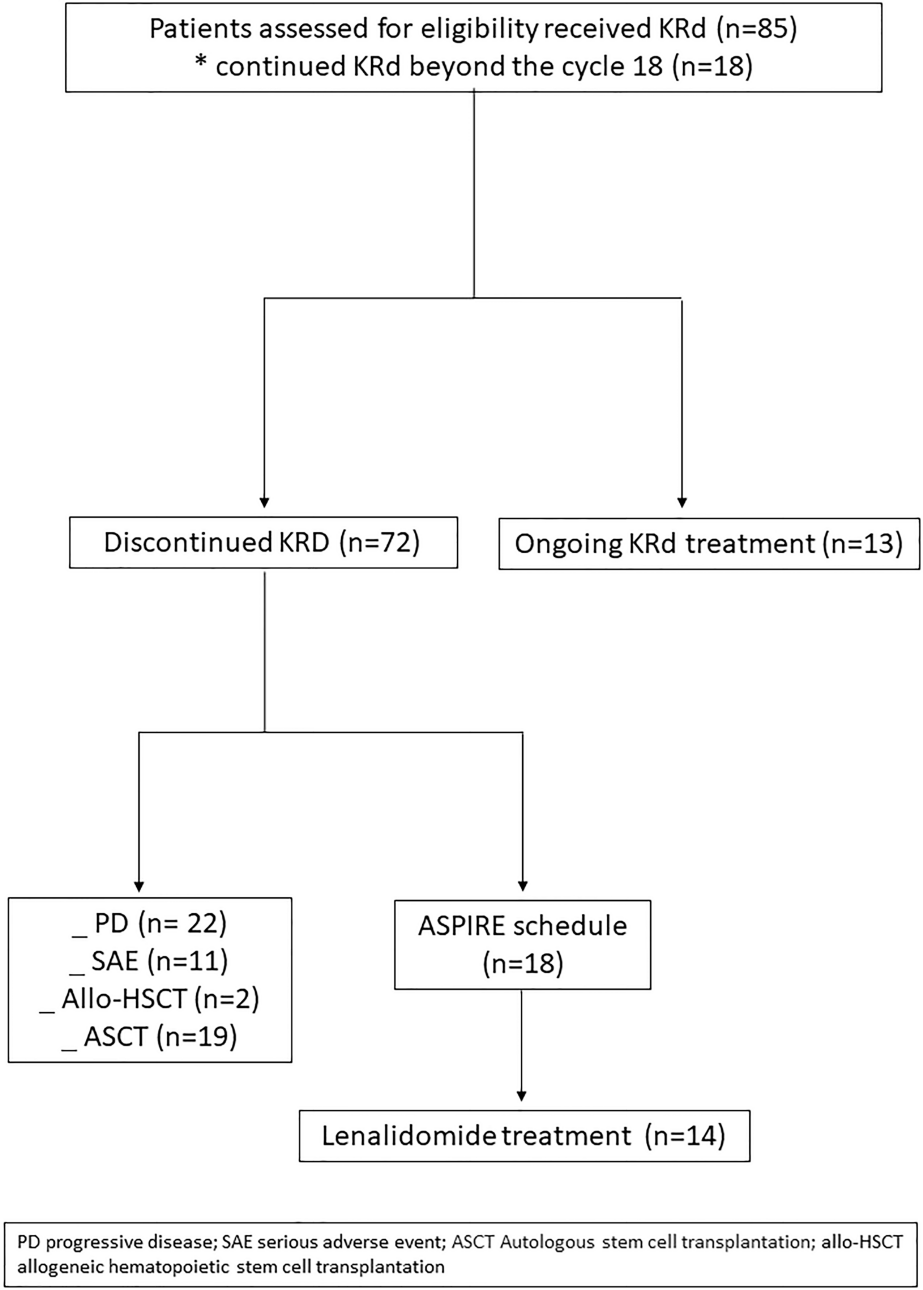
CONSORT flow diagram.

**Figure 2:**
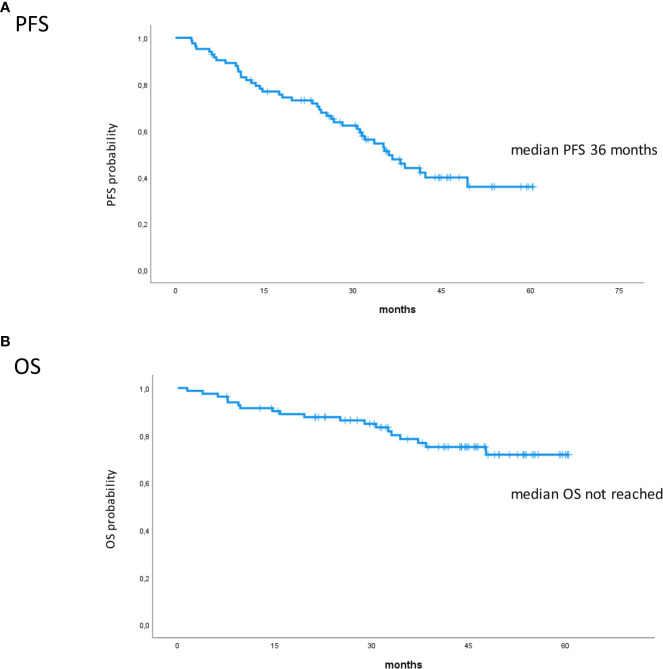
Kaplan–Meier survival curves. **(A)** PFS in all patients. **(B)** OS in all patients. PFS, progression-free survival; OS, overall survival.

**Figure 3:**
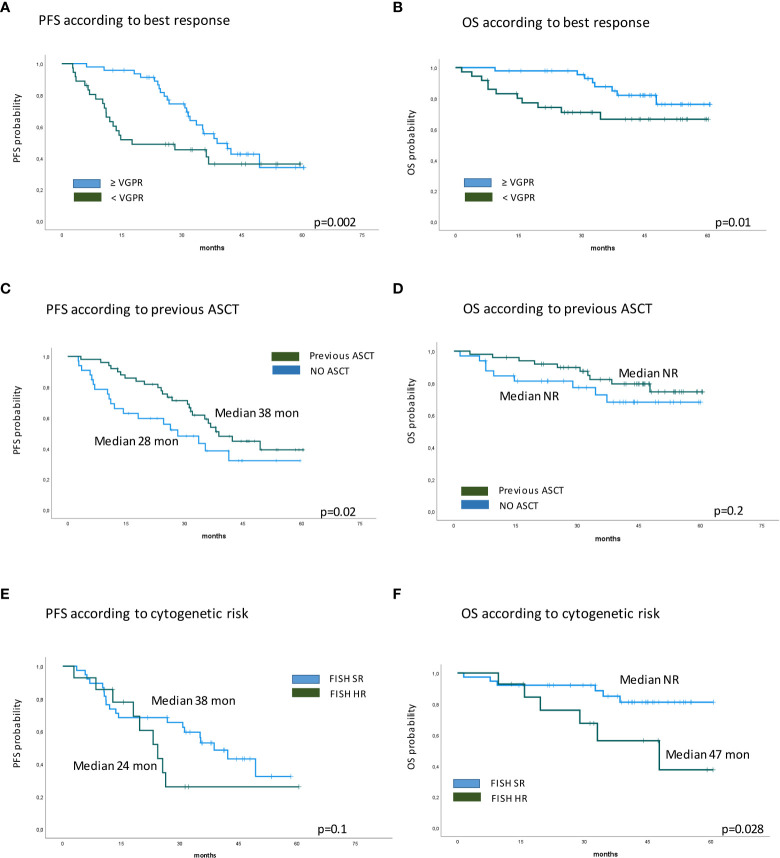
Kaplan–Meier survival curves. **(A)** PFS according to best response. **(B)** OS according to best response. **(C)** PFS according to previous ASCT. **(D)** OS according to previous ASCT. **(E)** PFS according to cytogenetic risk. **(F)** OS according to cytogenetic risk. PFS, progression-free survival; OS, overall survival; ASCT, autologous stem cell transplantation.

Eighty-five RRMM patients received KRd during the observation period. The characteristics of patients at the study entry are shown in **Table 1**. The median age of the patients in the analysis was 61 years (range 41–78); only five patients (6%) were older than 75 years. ISS was available in 62 (73%) patients: 24% and 26% of them were ISS-2 and ISS-3, respectively. High-risk cytogenetics was found in 26% of the patients. Sixty-two patients (73%) received KRd at the first relapse whereas 23 (27%) in the second relapse. Nearly all patients (98%) had received prior bortezomib-based treatment, and 37 (43%) were refractory to the last bortezomib-containing regimen. Nine patients (10.5%) were exposed to lenalidomide, with a refractory rate of 60%. A total of 36% of patients showed disease refractory to the last line of therapy, and 51 patients (60%) had received a previous autologous stem cell transplant (**Table 1**). After a median time from diagnosis of 28 months, the symptomatic disease was observed in 45 (53%) patients and a biochemical relapse in 40 (47%). Fifteen patients (17%) had a RI, being severe in six of them (7%) (eGFR < 30 ml/min), whereas 52 patients (62%) presented bone lesions highlighted by CT/PET or MRI. Three patients showed an extramedullary disease. Evaluation of the cardiological status at study entry revealed only one cardiovascular risk factor (hypertension, obesity, smoking, diabetes, dyslipidemia, and thromboembolic event) in 25 patients (29%) and at least two factors in 13 patients (15%). Baseline echocardiography was performed in 57 (67%) patients; median LVEF assessed at baseline was 60% (range 50–73); no cases of pre-existing cardiac amyloidosis were found. Administration of carfilzomib was initiated according to the treatment schedule in all patients, while the starting dose of lenalidomide was reduced in 13 patients (15%), based on renal function, and dexamethasone was adjusted in 12 patients (14%) to avoid alterations in glucose metabolism. After a median follow-up of 40 months, the median number of KRd courses administered was 16 (range 1–52). Two patients did not complete the first cycle because of cardiac toxicity and definitively discontinued the treatment; therefore, 83 patients were evaluable for response. The ORR was 95%. More in detail, stringent complete remission (sCR) and complete remission (CR were achieved in 8 patients (10%) and 23 patients (28%), respectively, and very good partial remission (VGPR) in 16 (19%) and partial remission (PR) in 32 patients (28%). The responses are summarized in **Table 2**. The best hematological response was observed after a median of 6 months (range 1–12 months) from the start of therapy. PET evaluation after 6 months revealed regression of known bone lesions in 90% of patients, while only one of the three patients with extramedullary disease had persistent PET-positive lesions after 6 months of therapy. Seventy-two patients (87%) discontinued the KRd combination: 22 patients (26%) due to disease progression, 21 (25%) according to physician decision (subsequent ASCT or allo-SCT), 11 (13%) due to toxicity or severe adverse events, and 18 (21%) according to the ASPIRE schedule, of whom 14 remained on lenalidomide (10–15 mg) after discontinuation of carfilzomib (**Figure 1**). Eighteen patients (21%) continued KRd treatment beyond the 18th cycle, and 13 patients (15%) were on ongoing treatment at the time of analysis (**Figure 1**). The median duration of treatment (DoT) was 18 months (range, 16.1–19.2 months); the type of relapse, the refractoriness to previous therapies, and the response obtained to KRd treatment did not have an impact on DoT. The median PFS was 36 months (range, 29.1–43.2 months; **Figure 2**). In our study cohort, the achievement of at least VGPR was associated with an improved PFS (median 38 *vs.* 17 months; HR = 1.73, 95% CI: 0.98–3.13, p = 0.002; **Figure 3A**). Patients with high-risk cytogenetic abnormalities showed a trend to lower PFS (median 24 *vs.* 39 months, p = 0.06; **Figure 3E**) compared to those with standard risk. RI, elevated lactate dehydrogenase (LDH), the number of prior lines of therapy, prior exposition/refractoriness to bortezomib, refractoriness of the last line of therapy, and type of relapse (biochemical or clinical) did not influence PFS (**Table 3**). Patients treated with ASCT (single or double) in the previous lines showed a longer PFS (median 38 *vs.* 28 months, HR = 0.59, 95% CI: 0.32–0.83, p = 0.02; **Figure 3C**). We also separately analyzed PFS for patients undergoing KRd in the first or second relapse or for refractoriness to the prior line, obtaining a median PFS of 49, 26, and 33 months, respectively (p = 0.02). During the observation period, 45 patients (52%) had a relapse requiring a subsequent line of treatment with a median time to next treatment (TTNT) of 36 months (range 32–40 months). The daratumumab–bortezomib–dexamethasone association or pomalidomide-based combinations were the most frequent treatments received by 42% and 22% of patients, respectively, resulting in a median PFS2 of 8 months (range, 1.7–15.5 months) and an ORR2 of 64%. Survival beyond progression was similar among treatment groups. At the cutoff date, 21 patients (25%) had died, mainly from PD (90%). The median OS was not reached (NR); the 5-year OS rate was 73% (**Figure 2**). An improved OS was observed for patients achieving ≥VGPR (p = 0.01); more precisely, a 30-month OS benefit for patients achieving ≥VGPR was 96% *vs.* 72% (HR = 2.3, 95% CI: 1.01–5.89, p = 0.01 **Figure 3B**). Patients with high-risk cytogenetic abnormalities showed a significantly shorter OS than those with standard risk (median 47 months *vs.* NR, HR = 3.3, 95% CI: 1.06–10.39, p = 0.028; **Figure 3F**). The other factors analyzed (type of relapse, line of therapy, type of therapy, and refractoriness to the last line of treatment) had no significant impact on OS (**Table 3**) (**Figure 3D**).

**Table 1:** Patients’ characteristics at KRd treatment.

Characteristics	Patients (N = 85)
Male/female	49/36
Median age at KRd start (years, range)	61 (41–78)
<75 years	80 (94%)
≥75 years	5 (6%)
Cytogenetic risk—no. of pts (%)	53 (62%)
Standard risk	39 (74%)
High risk	14 (26%)
ISS—no. of pts (%)	62 (73%)
I	31 (50%)
II	15 (24%)
III	16 (26%)
Type of myeloma—no. of pts (%)	
IgG	58 (68%)
IgA	14 (17%)
IgD	1 (1%)
Micromolecular	12 (12%)
Type of light chains—no. of pts (%)	
Kappa	52 (61%)
Lambda	33 (39%)
Renal impairment (eGFR < 30 ml/min)	6 (7%)
Previous lines of treatment—no. of patients (%)	
1 previous line	62 (73%)
2 previous lines	23 (27%)
Previous ASC—no. of patients (%)	51 (60%)
Refractory to last line of treatment	31 (36%)
Previous exposed	
Bortezomib	83 (98%)
Lenalidomide	9 (10.5%)
Disease refractory to bortezomib	37 (43.5%)
Disease refractory to lenalidomide	5 (60%)

KRd, carfilzomib in combination with lenalidomide and dexamethasone; ISS, International Staging System; eGFR, estimated glomerular filtration rate; ASC, autologous stem cell transplantation.

**Table 2:** Responses according to IMWG consensus criteria.

	Patients (N = 83)
Best responses—no. of pts (%)	
ORR	79 (95%)
CR	31 (38%)
VGPR	16 (19%)
PR	32 (38%)
MR-SD	3 (4%)
PD	1 (1%)
Median no. of cycles (range)	16 (1–52)

CR, complete remission; VGPR, very good partial response; PR, partial response; MR, minimal response; NR, stable disease; PD, progressive disease; IMWG, International Myeloma Working Group.

**Table 3:** Univariate analysis of PFS and OS.

	Univariate analysis					
	No. of pts	PFS			OS	
		Events		p	Events	p
Refractory to bortezomib				0.1		0.2
Yes	37	22			6	
No	46	22			13	
Previous ASCT				***0.02***		0.3
No	33	19			7	
Yes	50	25			10	
Refractory to last treatment				0.4		0.4
Yes	30	17			5	
No	53	27			14	
Type of relapse				0.3		0.2
Clinical relapse	45	27			13	
Biochemical relapse	38	17			6	
Previous lines of therapy				0.2		0.4
1	61	30			12	
2	22	14			7	
Response				***0.002***		***0.01***
>VGPR	47	23			8	
<VGPR	36	21			11	
Cytogenetics				0.06		***0.02***
SR	38	20			6	
HR	14	9			6	

PFS, progression-free survival; OS, overall survival; ASCT, autologous stem cell transplantation; VGPR, very good partial remission; SR, standard risk; HR, high risk.

Bold-italic values denotes statistically significant values.

### Safety

The KRd regimen was well tolerated, and the main toxicities observed are summarized in **Table 4**. The most common adverse events were hematological toxicities. Cardiovascular adverse events, gastrointestinal toxicity, thrombotic events, infections, and elevated liver function tests were the most common non-hematological toxicities. Grade 3 or higher anemia, thrombocytopenia, and neutropenia occurred in nine patients (11%), 18 patients (21%), and 24 patients (28%), respectively. The median time to onset of hematological toxicity was the third cycle. Among the cardiovascular events, the most frequent was arterial hypertension, which occurred in 10 patients (12%), while congestive heart failure, arrhythmia, and ischemic heart disease were less frequent and rarely grade 3–4. Seven patients developed deep vein thrombosis, and one patient developed grade 2 stroke. One patient died of acute pulmonary edema, which was not considered directly attributable to KRd treatment. At the beginning of the treatment, 25 patients reported cardiovascular risk factors, and among these, five (20%) developed a CVAE. Gastrointestinal adverse events occurred in 13 patients (15%) and mainly included diarrhea (76% of patients), whereas transaminase elevations (alanine and aspartate aminotransferase), thought to be secondary to carfilzomib, were observed in five (6%) patients, but always less than grade 3. Despite standard prophylaxis, infections of any grade were reported in 20 patients (24%), four patients (5%) experienced pneumonia, and one patient died of pulmonary infection (**Table 4**). During the study, three patients developed a COVID-19 infection, and one patient died from severe COVID-19 pneumonia. Nine patients (10%) required hospitalization for adverse events during therapy, mainly infections, and impaired renal function. Median hospitalization time was 11 days; all, except for the patient who died of COVID-19, resumed therapy at discharge. Carfilzomib dose reduction was reported for one patient due to hepatotoxicity, while two patients discontinued lenalidomide due to renal failure; lenalidomide dose reduction occurred in seven patients due to neutropenia and thrombocytopenia.

**Table 4:** Main toxicities (all grades and grade ≥ 3).

Adverse event	No. of patients (%)	
	Grade 3	Grade 4
Hematological		
Anemia	5 (6)	4 (5)
Thrombocytopenia	6 (7)	12 (14)
Neutropenia	18 (21)	6 (7)
	All grades	≥Grade 4
Non-hematological		
Gastrointestinal toxicities	13 (15)	–
Liver function test abnormal	5 (6)	–
Infections	15 (18)	5 (6)
Skin rash	5 (6)	–
Pyrexia	9 (10)	–
Peripheral neuropathy	4 (5)	–
Kidney injury	5 (6)	–
Muscle spasm	3 (3)	–
Fatigue	3 (3)	–
CVAE		
Hypertension	10 (12)	–
Arrhythmia	1 (1)	–
Cardiac failure	5 (6)	–
Ischemic heart disease	1	–
Thrombotic events	7 (8)	1 (2)

CVAE, cardiovascular adverse event.

### Bridge to transplant

Two patients were treated with KRd as re-induction therapy, followed by allogeneic transplantation, and both patients died: one from graft-versus-host disease (GVHD) and one from disease progression. Nineteen patients underwent KRd treatment as a bridge to autologous transplantation. Eight patients had already received a prior line of therapy (VTD) including an ASCT, and at relapse, the KRd combination was used as a re-induction (median 4–6 cycles) for a salvage ASCT. Eleven newly diagnosed patients were primary refractory to bortezomib-based regimens or had not achieved at least a good PR and therefore were not considered candidates for the transplant procedure. In this case, KRd was used to improve the response obtained to first-line treatment. Stem cell collection was performed with granulocyte colony-stimulating factor (G-CSF) alone or cyclophosphamide 3 g/m^2^ after 3–6 cycles of KRd; all patients were able to collect an adequate peripheral blood stem cell (PBSC) graft. The median number of KRd cycles administered before ASCT was 7 (range 3–8 cycles); three patients underwent double ASCT procedure, while four patients continued KRd after ASCT. Approximately half of the patients achieved a CR, 30% a VGPR, and 20% a PR. In almost all of the patients in CR and VGPR, the pre- and post-transplant minimal residual disease (MRD) was evaluated using next-generation flow cytometry, obtaining negativity in 65% of cases.

## Discussion

Recent advances in the treatment of MM led to improvements in depth of response, PFS, and OS. However, MM remains an incurable disease characterized by a recurring pattern of relapse and remission. Carfilzomib is a potent, irreversible, selective proteasome inhibitor that showed robust activity in myeloma, both as a single agent and in combination with other anti-myeloma agents (1–4, 20, 21). In the ASPIRE trial, carfilzomib in combination with lenalidomide led to a deep quality of response and high overall response rate in the RRMM subset, with prolongation of PFS and OS. Several retrospective studies reported information regarding the efficacy and tolerability of KRd treatment, even in frail patients (i.e., renal insufficiency or amyloidosis) typically excluded from clinical trials. Prospective data obtained from real life, directly comparable with the ASPIRE study, which allowed us to analyze responses to treatment even in the long term in patients in the early stages of the disease, are still lacking. To the best of our knowledge, this is the first prospective observational study analyzing the impact of KRd in two or three LOTs, started in December 2016, as soon as carfilzomib was made commercially available in Italy, with a duration of follow-up of 5 years. The study population had a mean age of 61 years, not much different from ASPIRE patients. It should be noted that only 6% of our patients were over 75 years of age at the onset of KRd because initially, to avoid the reported cardiovascular toxicities, this type of combination was mainly offered to younger patients. Unlike ASPIRE, our series includes patients with extramedullary disease, with a higher incidence of high-risk cytogenetics abnormalities (26% *vs.* ASPIRE 12%) and with impaired renal function (17% *vs.* ASPIRE 6%, eGFR < 30 ml/min in 7% *vs.* 0% in ASPIRE). In our study ORR (95%) and CR rate (38%) were unexpectedly higher than in ASPIRE (87% and 32%, respectively), probably because most of our patients were in the first relapse (70%) when compared to ASPIRE in which most patients had already undergone two to three previous regimens. Similar to the ASPIRE study, prior use of bortezomib did not influence the efficacy of KRd treatment in terms of outcome: nearly all of our patients had been exposed to bortezomib with a refractoriness rate of 43% (22).

The median DoT was 18 months, which represents the duration of therapy according to the ASPIRE schedule, although approximately 20% of our patients continued the therapy beyond 18 cycles, in the absence of toxicity. We found that PFS and TTNT (both 36 months) were better than described in the literature, confirming also in our analysis that obtaining good quality responses has a significant impact on PFS (6). These data can be supported by several considerations: most patients were in LOT 2; some patients maintained therapy for more than 18 cycles or consolidated KRd with a transplant procedure. PET evaluation after 6 months of therapy, with negativization of metabolically active lesions including extramedullary disease in the majority of patients, adds further information on the efficacy of this therapeutic regimen in aggressive relapses. In our study, after a median follow-up of 40 months, the median OS was not reached; however, the 60-month analysis showed a survival rate of 73%. Furthermore, supporting the results of the prospectively planned final analysis of the ASPIRE study, after a median follow-up of 67.1 months, a median OS of 48.3 months was found in the KRd group (23). Significantly shorter survival outcomes were shown for patients with high-risk cytogenetics (OS 47 months, p = 0.028), confirming that KRd improves but does not abrogate the worse prognosis associated with cytogenetic abnormalities. In a post-hoc analysis of the ASPIRE study (7), KRd improved PFS and ORR regardless of prior transplant procedure; also in our study, the carfilzomib-based treatment led to an improvement in PFS (median 38 *vs.* 28 months, p = 0.02; **Figure 2**) in patients who relapsed after a previous ASCT. Furthermore, our data also confirmed the efficacy of KRd as a bridge to ASCT; despite the presence of primary refractory patients, we obtained excellent ORR values with CR percentages of 50% and MRD negativity of 66%. Our data confirmed also the feasibility and safety of the KRd regimen in real life, with a low discontinuation rate (6%). The most common grade ≥3 hematologic and non-hematologic adverse events were neutropenia (21%), thrombocytopenia (7%), cardiac failure (6%), and infections (10%). Notably, these results confirm some previously published data reporting a similar incidence of adverse events in carfilzomib-based triplets, including pneumonia, respiratory tract infection, and pyrexia (24). Our data are consistent with the ASPIRE study results, major adverse events were hematologic, and most of them were well managed by reducing the lenalidomide dose. We also noted a high incidence of neutropenia and infectious complications such as pneumonia, despite adequate antibiotic and antiviral prophylaxes.

We acknowledge as major limitations of our study the small sample size and the lack of data on minimal residual disease including non-transplant patients who achieved at least a VGPR. Despite these pitfalls, this is the largest prospective study to date outside the clinical trial setting. Our data confirm that this therapeutic modality is effective and relatively safe in the early stages of the disease and can be used as a re-induction for a salvage autologous transplant in a real-world setting. A longer follow-up is obviously needed to also confirm the benefit of the KRd combination after 18 cycles.

## Data availability statement

The raw data supporting the conclusions of this article will be made available by the authors, without undue reservation.

## Ethics statement

The studies involving human participants were reviewed and approved by Comitato Etico, AOU careggi hospital, Florence Italy. The patients/participants provided their written informed consent to participate in this study.

## Author contributions

EA was responsible for the study design, collected the clinical data, and wrote the report. IA, VC, FS, MD, SC, MS, and SGr collected the clinical data. SP and IA were responsible for the analysis and interpretation of data. AB and MM were responsible for the clinical trial management. AG, GB, MB, SGa, and AV contributed to the critical review and finalized the report. All authors contributed to the article and approved the submitted version.
